# Validation of Diet ID™ in Predicting Nutrient Intake Compared to Dietary Recalls, Skin Carotenoid Scores, and Plasma Carotenoids in University Students

**DOI:** 10.3390/nu15020409

**Published:** 2023-01-13

**Authors:** Marcela D. Radtke, Gwen M. Chodur, Michael C. S. Bissell, Leslie C. Kemp, Valentina Medici, Francene M. Steinberg, Rachel E. Scherr

**Affiliations:** 1 Department of Nutrition, University of California, Davis, CA 95616, USA; 2 Center for Nutrition in Schools, University of California, Davis, CA 95616, USA; 3 Aggie Compass, Office of Student Affairs, University of California, Davis, CA 95616, USA; 4 Division of Biostatistics, Department of Public Health Sciences, University of California Davis School of Medicine, Davis, CA 95616, USA; 5 Department of Internal Medicine, Division of Gastroenterology and Hepatology, University of California, Davis, CA 95616, USA

**Keywords:** Diet ID™, diet quality photo navigation, dietary assessment, diet patterns, nutrient intake, college students, NDSR, Veggie Meter^®^

## Abstract

**Background and Aim:** Collecting accurate dietary information in the research setting is challenging due to the inherent biases, duration, and resource-intensive nature of traditional data collection methods. Diet ID™ is a novel, rapid assessment method that uses an image-based algorithm to identify dietary patterns and estimate nutrient intake. The purpose of this analysis was to explore the criterion validity between Diet ID™ and additional measures of dietary intake. **Methods:** This prospective cohort study (*n* = 42) collected dietary information using Diet ID™, the Nutrition Data System for Research (NDSR), plasma carotenoid concentrations, and the Veggie Meter^®^ to estimate carotenoid levels in the skin. **Results:** There were significant correlations between Diet ID™ and NDSR for diet quality, calories, carbohydrates, protein, fiber, and cholesterol. Vitamin A and carotenoid intake were significantly correlated, with the exception of α-carotene and lycopene. Significant correlations were observed for calcium, folate, iron, sodium, potassium, Vitamins B_2_, B_3_, B_6_, C, and E. Skin carotenoid scores and plasma carotenoids were correlated with carotenoid intake from Diet ID™. **Conclusions:** Diet ID™ may be a useful tool in nutrition research as a less time-intensive and minimally burdensome dietary data collection method for both participants and researchers.

## 1. Introduction

Collecting accurate information on dietary intake is an essential component of understanding the physiological relationship between food and health [1,2]. In particular, the habitual consumption of fruits and vegetables is associated with improved biomarkers for health and the reduction of chronic disease risk across the lifespan due to the vitamins, minerals, phytonutrients, fiber, and other bioactive compounds [3,4]. Commonly used measures of fruit and vegetable intake include 24 h dietary recalls, food frequency questionnaires (FFQs), and food records [5,6]. These subjective assessment tools often introduce unintended reporting errors or response biases that may impact the accuracy of dietary data [7]. Objective measures may also be implemented to determine nutrient consumption, such as blood or urinary biomarkers, and tissue or dermal biopsies [6]. However, such assessments are inherently resource-intensive and subject to participant and researcher burden [8]. Therefore, innovative techniques for rigorously assessing dietary intake, emphasizing fruit and vegetable consumption, are warranted in the research setting [9].

Carotenoids are a class of phytochemicals found in many fruits and vegetables and therefore are a useful marker for dietary assessment. Carotenoids are fat-soluble compounds that are transported in lipoproteins, making them detectable and quantifiable in the blood and skin [10]. In addition to identifying dietary carotenoids through traditional dietary assessments, carotenoid levels may also be identified through innovative techniques, such as spectroscopy-based skin carotenoid measurements and technology-based or image-based dietary assessment methods [11,12,13]. The Veggie Meter^®^ is a device that utilizes pressure-mediated reflection spectroscopy to quantify the density of carotenoids in the skin [14]. Skin carotenoid scores (SCS) are reflective of long-term dietary changes, approximately one month of intake, due to the longer half-life and slower degradation of carotenoids in the skin compared to plasma or serum, which is evident of approximately two weeks of dietary intake [15,16]. Technology-based or image-based dietary assessment methods may have the capacity to evaluate both short- and long-term dietary intake of carotenoid compounds.

Photo navigation technology is an emerging approach used to estimate dietary patterns and nutrient intake in the research setting. The transition from static images of dietary intake using cameras or handheld devices to dynamic, real-time image-assisted or image-based dietary technologies provides additional improvements for mitigating common errors and biases in traditional dietary assessments [17]. Validation studies comparing image-based technologies to other forms of dietary assessments, including 24 h dietary recalls, weighed food records, and double-labeled water, found inconsistencies between the methods of reporting dietary intake, further highlighting the need for the development of more accurate and reliable image-based dietary assessment tools [18]. In addition to the limited number of validated image-based dietary assessment techniques, most studies have yet to include micronutrients, phytonutrients, or other bioactive compounds, making it challenging to definitively quantify the prominent components of fruit and vegetable intake using such methods [18].

Diet ID™ is a novel application that assesses dietary patterns through Diet Quality Photo Navigation (DQPN^®^), a patented image-based algorithm that provides estimates of nutrient intake, based on a series of food images [19,20]. Diet ID™ was developed using dietary data extracted from the National Health and Nutrition Examination Survey (NHANES), as well as a comprehensive review of food intake surveys and epidemiological research to determine estimates of dietary patterns, portion sizes, and eating frequencies of adults in the United States (US) [19,20]. Diet ID™ provides nutrient estimations for energy intake, macronutrients, and micronutrients, including phytonutrients and other bioactive compounds, such as carotenoids based on the NDSR food database. Diet ID™ not only estimates total carotenoid intake but quantifies the nutrient output for the following carotenoid compounds: α-carotene, β-carotene, lycopene, lutein, and zeaxanthin. Research exploring the relationship between individual carotenoid compounds and total carotenoid intake estimated by Diet ID™ with other measures of fruit and vegetable intake has yet to be conducted.

The present analysis aimed to explore the criterion validity of Diet ID™ against other methods of dietary assessments, including plasma carotenoid concentrations, skin carotenoid scores, and 24 h dietary recalls in a population of university students. This validation study was derived from a larger study that seeks to investigate various biomarkers found in blood and skin, and to measure dietary intake through repeated 24 h NDSR recalls and Diet ID™ to determine if food access programs at the University of California, Davis improve biomarkers for health and fruit and vegetable consumption among students who use these services.

## 2. Materials and Methods

The protocol and procedures for this study were approved by the University of California, Davis Institutional Review Board. Participants provided informed written consent prior to study commencement (1476178-4).

### 2.1. Study Design

A prospective cohort (*n* = 42) consisting of college students from the University of California, Davis was recruited in January 2020 to participate in an effectiveness evaluation of campus food access programs. The study timeline was selected to minimize excessive sun exposure, reduce the variation from seasonal, high carotenoid-containing foods, such as squash, tomatoes, and berries [21,22], and for winter break to serve as a washout period for students who had used campus food access programs prior to enrolling in the study. The study duration was conducted in accordance with the 10-week academic quarter (January–March 2020), with the first data collection period occurring during weeks 1–3 and the second data collection period occurring in weeks 8–10 of the term. Specific to the larger evaluation study, an eight-week duration between timepoints was allotted to ensure biomarkers of interest had an adequate acclimation period to respond to changes in dietary intake.

Participants were recruited prospectively through fliers, social media, and other means of communication, such as verbal or email contact. Participants were healthy, biological males and females above the age of 18 currently enrolled as undergraduate or graduate students at the University of California, Davis, and within a BMI range of 18.5–34.9 kg/m^2^ [23,24]. Exclusion criteria included smoking or living in a household with an indoor smoker (including cigarettes, electronic cigarettes, vaping, marijuana), consuming edible products containing tetrahydrocannabinol (THC), the psychoactive component in marijuana, and excessive drinking (consuming >5 alcoholic drinks per week), as the metabolism and absorption of carotenoid compounds under these conditions is unknown [10]. Additionally, individuals participating in artificial tanning methods, such as UV light exposure, or consuming oral or topical high-dose Vitamin A medication (i.e., Accutane, retinol cream) were ineligible to participate due to the potential for elevated carotenoid detection in the blood or skin from non-dietary sources [25]. Prospective study subjects completed a short screening by telephone and those who met the inclusion criteria were invited to schedule an in-person study visit. Study visits were conducted at the Ragle Human Nutrition Research Center at the University of California, Davis.

### 2.2. Anthropometric Data

Anthropometric data were collected at each timepoint to capture any changes during the study period. Height and weight were measured twice to ensure values were within 0.3 cm and 0.1 kg, respectively, and the mean value was reported. Height was measured using a stadiometer and weight was measured using a digital scale; subsequently, BMI was calculated as weight in kilograms divided by height in meters squared (kg/m^2^). Blood pressure was measured twice with a sphygmomanometer for an average reading, to ensure participants were normotensive.

### 2.3. Sociodemographic Data

Sociodemographic information including age, sex, race/ethnicity, food security status, and physical activity was acquired for inclusion as potential covariates. Participants self-reported use of food access resources. Food security status was measured at both study timepoints using the United States Department of Agriculture (USDA) 10-item Adult Food Security Survey Module [26]. The following classifications were used in accordance with the USDA to indicate food security status over the last 30 days: 0: high food security; 1–2: marginal food security; 3–5: low food security; and 6–10: very low food security [27].

### 2.4. Dietary Intake Data

#### 2.4.1. Diet ID™

Participants completed the Diet ID™ assessment in person at each clinic visit. As the application is designed to measure habitual dietary patterns over the last 30 days of intake, only one assessment per timepoint was required. Participants received detailed instructions provided by the manufacturer for standardization among users.

Diet ID™ initially provided a set of screening questions to identify select food group consumption, such as a vegan, vegetarian, gluten-free, or alcohol-free dietary patterns. The application then displayed two images containing a variety of food items to identify the general types of foods that may be consumed. As the users selected the food items most similar to those they consume regularly, the algorithm provided more specific images by incorporating varying types of the same foods, such as low-fat versus full-fat dairy products, and asked individuals to choose the food images that may be present in their eating pattern on a day-to-day basis. Once the application identified an individual’s typical eating pattern, foods from the final image were quantified for nutrient analysis by the Diet ID™ algorithm in accordance with the Nutrition Data System for Research (NDSR) database (Version 2017). In addition to specific nutrient output, diet quality was computed by Diet ID™ software using criteria from the Healthy Eating Index 2015 (HEI-2015) [28]. When participants completed multiple Diet ID™ assessments at the same study visit, the nutrient values from first assessment were used.

#### 2.4.2. NDSR 24 h Dietary Recalls

Three 24 h dietary recalls using NDSR Software (Version 2019) were conducted by phone within one week of each in-person clinic visit, for a total of six recalls per participant. Each recall (*n* = 252) was unannounced and consisted of two non-consecutive weekdays (*n* = 180) and one weekend day (*n* = 72), when possible, to capture potential variations in dietary intake and to minimize observer bias. Participants who did not respond to researcher inquiries over the weekend had all recalls recorded on weekdays to ensure three days of intake were collected within a week of the in-person clinic visit. Dietary recalls were conducted by trained researchers under the guidance of a registered dietitian. Participants were asked to report all intake starting from midnight the previous day, inclusive of food, beverages, and supplements. As quality control, the supervising registered dietitian compared the intake as entered in the initial “Quick List” to the “Food Record.” Due to the racial and ethnic diversity of the sample population, some of the culturally diverse foods consumed were not matched to records in the NDSR database. Missing food items were reviewed independently by two researchers for consistency with other records in the database. Examples of food classified as “missing” from the NDSR database included boba or bubble tea, international snacks (i.e., shrimp chips, fish jerky) and brand-specific items (i.e., Kirkland protein bars, Dave’s Killer Bread). Food labels were reviewed for nutrient analysis if no best fit in the NDSR system was identified. Diet quality, measured using the HEI-2015, was calculated based on the nine components of nutrient adequacy and the four components of nutrient moderation from the foods consumed in the NDSR dietary recalls [29]. Total carotenoids were calculated through summation of individual carotenoid output from NDSR in micrograms (mcg).

### 2.5. Skin Carotenoid Scores

Skin carotenoid scores were measured using the Veggie Meter^®^. The Veggie Meter^®^ is a validated, research-grade instrument that utilizes pressure-mediated reflection spectroscopy to estimate carotenoid concentration in the skin [11]. The protocol for collecting data using the Veggie Meter^®^, including triplicate measures and the use of the non-dominant ring finger, was followed to ensure that inter- and intra-individual variability, as well as environmental interferences, were minimized [30].

### 2.6. Plasma Carotenoids

Participants were asked to abstain from food and beverages, excluding water, for a minimum of 10 h prior to the study visit. Blood samples were collected through venipuncture by a trained phlebotomist at the Ragle Human Nutrition Research Center using EDTA vacutainer blood collection tubes. Whole blood was centrifuged at 1500 rpm for 15 min at 4 °C and the plasma was extracted, aliquoted, and stored at −80 °C prior to carotenoid analysis performed by Eurofins Craft Technologies.

Individual carotenoids were measured by HPLC in plasma using a modification of the procedures described by Craft [31,32]. Briefly, after thawing, 150 μL aliquots of plasma were diluted with 150 μL of water containing 0.01% ascorbic acid and 0.001% EDTA then deproteinated by vortexing with 300 μL of ethanol containing tocol as an internal standard and butylated hydroxytoluene (250 ppm) as an antioxidant. The samples were extracted by vortex mixing for 2 min with 2 mL of hexane. Samples were centrifuged to separate phases and the upper hexane was transferred to a borosilicate tube. The extraction was repeated. The combined supernatant was evaporated using a centrifugal evaporator. The residue was dissolved with vortex mixing in 30 μL of ethyl acetate then diluted with 100 μL of acetonitrile:isopropanol (9:1) and vortex mixed 15 s prior to placement in the autosampler. A 20 μL volume was injected.

The HPLC system consisted of a Chromeleon data system, a solvent degasser, an autosampler maintaining samples at 20 °C, a Polaris C18 Ether (3 μm, 4.0 mm × 250 mm), a guard column containing similar stationary phase, a column heater at 31 °C, a diode array detector to measure carotenoids at 450 nm, 325 nm, and at 295 nm to measure tocol. The separation was performed isocratically using a mobile phase of 83% acetonitrile/13% dioxane/4% methanol containing 150 mM ammonium acetate and 0.1% triethylamine at a flow rate of 1.0 mL for 21 min. The method is calibrated with neat standards within the physiological range which are assigned concentrations using absorption coefficients (E1% cm) and corrected for HPLC purity [33]. The calibration method is based on external standards using peak areas and corrected for tocol as the internal standard.

### 2.7. Statistical Analysis

Data were inspected for normality using Shapiro–Wilk test and transformed as necessary. Descriptive data on participant characteristics are expressed as mean ± SD or percentage. Nutrient analysis from each set of three 24 h NDSR dietary recalls were averaged for a single mean output. Healthy Eating Index 2015 scores were computed from NDSR output using SAS code provided by the NDSR manufacturers. The SAS version 9.4 statistical software was used (SAS Institute Inc.) [34]. Paired *t*-tests were used to determine if dietary intake was independent at each of the timepoints. Considering dietary intake was not independent by timepoint, data from both study timepoints were averaged to determine the relationship between Diet ID™ and NDSR 24 h dietary recalls. Pearson’s correlations were computed to explore associations between the nutrients estimated by the dietary assessment instruments. Kendall’s tau was computed for variables with a non-linear relationship (HEI-2015) and those with distributions that did not conform to normality after transformation (cholesterol, Vitamin B_12_, α-carotene, β-carotene, and lycopene). Bland–Altman Plot Analysis was also performed to characterize the agreement between Diet ID™ and NDSR [35]. Nutrients of interest for this analysis were selected based on existing literature from dietary intake studies with the objective of comparing nutrient consumption to other biomarkers of dietary intake [36,37,38,39]. Also included were nutrients of concern for underconsumption (calcium, potassium, fiber, and vitamin D) as defined by the 2020–2025 USDA Dietary Guidelines for Americans [40]. Linear regression models were used to estimate the association between Diet ID™, skin carotenoid scores, and plasma carotenoids controlling for BMI, as previous research has demonstrated inverse correlations between BMI and carotenoid concentrations [41,42,43]. The vce(robust) command was used to obtain the robust estimator of variance in linear regression models that did not conform to assumptions of homoscedasticity. Statistical significance was established at *p* < 0.05. All other statistical analyses were performed using STATA Version 16 [44].

As this criterion-related validation study is a subset from a larger study, the sample size was initially computed a priori with the primary objective of comparing plasma carotenoids to skin carotenoid scores [12]. A post-hoc analysis for a minimal detectable difference was calculated to determine the number of participants needed to compare Diet ID™ against 24 h NDSR dietary recalls based on α = 0.05 and 80% power, in which a minimum of 30 participants were required [36]. As Diet ID™ is a novel assessment tool, additional studies comparing NDSR with other innovative dietary assessment methods were used as comparisons to determine the minimal detectable difference, confirming that the number of participants in this analysis surpasses the number of participants in previous studies that were sufficiently powered [45,46].

## 3. Results

### 3.1. Participant Characteristics

A total of 48 participants completed the baseline visit of the study, with six participants unable or unwilling to complete the second timepoint; therefore, 42 participants completed timepoint two and are included in the present analysis. Baseline characteristics of the study population are presented in Table 1. The cohort was 75% female, with a mean age of 22.09 ± 2.36 years and BMI of 24.58 ± 5.04 kg/m^2^. Of the total participants, 40% were categorized as having high food security, 31% had marginal food security, 17% had low food security, and 12% had very low food security. Results from a paired *t*-test found no significant changes in skin carotenoid scores from timepoint one to timepoint two, with average scores of 322.98 ± 114.42 and 341.35 ± 113.98, respectively (*p* = 0.38). Participants completed Diet ID™ in 3.68 ± 2.04 min.

### 3.2. Diet ID™ and 24 h NDSR Dietary Recalls

The average nutrient intakes from three 24 h NDSR recalls were significantly correlated with the findings from Diet ID™ for nearly all nutrients evaluated (Table 2). Diet quality was assessed in accordance with HEI-2015, using the nutrient criteria for adequacy and moderation from both dietary intake assessment methods. A significant correlation was observed for diet quality using HEI-2015 as estimated by 24 h NDSR dietary recalls and Diet ID™ (τ = 0.55, *p* < 0.0001). Total calories (kcals), protein intake, and carbohydrate intake were significantly correlated between the two instruments (*ρ* = 0.36, *p* = 0.02; *ρ* = 0.55, *p* = 0.002; *ρ* = 0.31, *p* = 0.05 respectively); however, there was not a significant correlation between the two instruments’ measurement of fat intake. To further explore the relationship between different nutrient subtypes for carbohydrates and fat intake as estimated by Diet ID™ and 24 h NDSR dietary recalls, dietary fiber and cholesterol were independently assessed. Significant associations were observed in measurements of dietary fiber (*ρ* = 0.64, *p* < 0.0001, as well as cholesterol (τ = 0.32, *p* = 0.003).

Of specific interest to the study was the consumption of Vitamin A, carotenoids, and carotenoid derivatives. There was a significant correlation of both Vitamin A (*ρ* = 0.39, *p* = 0.01) and total dietary carotenoid intake (*ρ* = 0.44, *p* = 0.003) between Diet ID™ and 24 h NDSR dietary recalls (Table 2). Significant associations were observed regarding the intake of individual carotenoids, including β-carotene (τ = 0.39, *p* = 0.0003), zeaxanthin, and lutein (*ρ* = 0.58, *p* = 0.0001), apart from lycopene (τ = −0.09, *p* = 0.40) and α-carotene, which was approaching significance (τ = 0.14, *p* = 0.19). Additionally, calcium, potassium, folate, iron, sodium, Vitamins B_2_, B_3_, B_6_, C, and E were significantly correlated, with the exception of Vitamins D, B_1_, and B_12_.

Bland–Altman Plots were generated to characterize the agreement between Diet ID™ and 24 h NDSR dietary recalls for all nutrients of interest. For all nutrients of interest, a majority with the data points fell within the 95% CI, with a maximum of three individuals out of the 42 participants in the sample not within the limits of agreement, with the exception of sodium (*n* = 5) (Supplemental File S1).

### 3.3. Diet ID™, Skin Carotenoid Scores, and Plasma Carotenoids

Diet ID™, skin carotenoid scores, and plasma carotenoids were compared to determine if objective concentration biomarkers of dietary intake were associated with nutrient estimations from Diet ID™ (Table 3). Total carotenoid intake measured by Diet ID™ was significantly correlated with skin carotenoid scores from the Veggie Meter^®^ after controlling for BMI (Adjusted R^2^ = 0.41, *p* < 0.0001). Significant positive associations were observed between total plasma carotenoids and total carotenoids estimated by Diet ID™, when controlling for BMI (Adjusted R^2^ = 0.37, *p* = 0.0001). To directly compare the objective measures of dietary intake, skin carotenoid scores and plasma carotenoids were assessed, and a strong positive correlation was observed after controlling for BMI (Adjusted R^2^ = 0.68; *p* < 0.0001).

## 4. Discussion

Diet ID™ was designed to assess dietary patterns and estimate nutrient intake values by means of a unique pattern recognition image-based algorithm to ultimately identify chronic disease risk [20]. This analysis demonstrates that nutrient intake from Diet ID™ was comparable to both short-term nutrient consumption from NDSR dietary recalls and plasma carotenoids, in addition to more long-term dietary intake determined by skin carotenoid scores. Diet ID™ was effective in estimating diet quality, as well as nutrients and bioactive compounds associated with fruit and vegetable consumption.

### 4.1. Total Calories and Macronutrients

Total calorie intake is an important nutritional marker used to estimate energy balance and is often pertinent in guiding nutrient recommendations and in nutrition research studies assessing weight gain or weight loss [47]. Total calorie, protein, and carbohydrate intake from Diet ID™ was associated with NDSR output. Although the measurement of total fat was not significantly correlated between Diet ID™ and NDSR, dietary fiber and cholesterol were both found to have significant associations between instruments. As measurements of fat intake approaches significance (*ρ* = 0.29, *p* = 0.06), the sample may have been limited in power to detect the criterion validity between devices for this macronutrient. It is important to note that the Diet ID™ software asks participants to report any dietary restrictions prior to the assessment. Thirteen participants indicated that they did not consume one or more of the following: eggs, nuts, dairy, or meat, which may provide insight into the discrepancies in fat consumption, as the images from Diet ID™ may have not accurately captured additional dietary sources of this macronutrient. Research looking at the dietary intake in the college student population has confirmed the challenges in self-reporting macronutrient intake using innovative technology [48]; further exploration into assessing the discordance in consumption is warranted.

Previous research on dietary intake data collection has indicated inconsistencies between subjective reporting of macronutrient consumption compared to objective measures of macronutrient intake [7,49,50]. It has been observed that individuals often underestimate the portion sizes of foods containing both protein and fat, as these are often measured in dietary data collection using weight estimations, which can be challenging to infer [51]. Additional demographic factors have been observed to introduce a higher risk of bias into the reporting of macronutrient intake, with females, individuals who are overweight or obese, individuals of low socioeconomic status, and individuals actively seeking to lose weight often underreporting macronutrients, whereas younger individuals and individuals with lower BMIs overestimating macronutrient consumption [52,53]. With the racial and ethnic diversity of the study population, including differences in socioeconomic status indicated by food insecurity, BMI, and a majority of the participants being biologically female, the increased likelihood of reporting bias with macronutrient intake may provide further insight to the non-significant finding for fat intake.

### 4.2. Micronutrients and Phytonutrients

Measurements of Vitamin A, Vitamin C, Vitamin E, Vitamin B_2_ (riboflavin), Vitamin B_3_ (niacin), Vitamin B_6_ (pyridoxine), folate, iron, sodium, potassium, carotenoids, and carotenoid derivative intakes as predicted by Diet ID™ were significantly correlated with NDSR output, whereas lycopene, α-carotene, Vitamin D, Vitamin B_1_ (thiamin), and Vitamin B_12_ (cobalamin) were not. Lycopene has been reported to be a challenging carotenoid to measure using traditional dietary assessment tools due to the considerable variability in degradation kinetics, dependent on processing and competing nutrient interactions within the food matrix [54,55]. It has been observed that lycopene bioavailability is higher in its cooked form compared to raw form, and therefore concentrations may differ depending on the preparation of lycopene-containing foods [56]. Lycopene metabolism and absorption has been shown to be highly correlated and contingent on macronutrient intake, specifically dietary fat and oil consumption [57]. Dietary sources of lycopene are in the *all-trans* configuration, which differs from the lycopene found in human tissue, which is in the cis-isomer configuration [58]. Due to the bulkiness of the all-trans lycopene, there is a lower affinity and efficiency for micelle incorporation, and therefore higher amounts of dietary fat may inhibit the absorption of lycopene [58]. This contradicts the physiological uptake of other carotenoid compounds, in which absorption and bioavailability increases with dietary fat consumption [59]. It is unknown whether correcting for the processing of lycopene-containing foods would alter the estimated nutrient values from Diet ID™ and 24 h NDSR dietary recalls. Additionally, lycopene is predominantly present in tomatoes and tomato-based products, limiting the availability of lycopene intake from food sources, whereas other carotenoids, such as β-carotene, are found more ubiquitously in red, orange, yellow, purple, and dark green foods [60].

Skin carotenoid scores and plasma carotenoids were used as objective measures of fruit and vegetable consumption. As overweight and obesity impacts the storage capacity of carotenoids in circulation, as well as those deposited in the skin, BMI was added as a covariate into the statistical model. Significant associations were observed between plasma carotenoids, skin carotenoid scores as measured by the Veggie Meter^®^, and dietary intake of total carotenoids as predicted by Diet ID™. The relationship between dietary intake and skin carotenoid scores is to be expected, as skin carotenoids represent a longer-term dietary intake of carotenoid-containing fruits and vegetables and therefore may be influenced by accretion [15]. Previous research using objective measures of dietary intake, such as plasma carotenoids or spectroscopy-based skin carotenoid measurements have also demonstrated similar moderate or weak associations due to discrepancies between subjective assessment tools for fruit and vegetable consumption and objective skin carotenoid scores [21,61]. The observed association highlights the use of Diet ID™ as an estimate for fruit and vegetable consumption and provides the capability to extrapolate nutrient values that are comparable to carotenoid concentrations detected in plasma and skin; however, it should be acknowledged that Diet ID™ may have limited utility as a dietary assessment tool as this comparison has only been demonstrated for carotenoid consumption in a US population.

Vitamin D intake was not significantly correlated between dietary assessment instruments. As an identified 2020–2025 DGA Nutrient of Concern, Vitamin D is only found in a small number of dietary sources, making nutrient adequacy challenging to achieve. The variation in the database from the 2017 version of NDSR and the 2019 version may explain the non-significant correlation, which likely was a result of Diet ID™ not including any fortified food items into the DQPN algorithm, such as Vitamin D found in fortified dairy products, cereals, and juices [62,63]. Similarly, enriched and fortified grain products are a main dietary source of thiamin and therefore may have not been accurately captured by the DQPN algorithm [64,65]. When participants were asked about fortified food products during the repeated NDSR dietary recalls, fortification status was often unknown and thus NDSR defaults were used for computation. The difficulty accounting for nutrients naturally found in a limited number of food items and intake of fortified foods may explain the deviation between instruments for Vitamin D and thiamin intake [66].

Due to the dietary restrictions reported by participants, specifically relating to the lack animal-based food products such as eggs, meat, and lactose intolerance, consumption of overall Vitamin B_12_ intake may have been inaccurately captured. It has been previously observed that individuals following a vegetarian or vegan dietary pattern are at an increased risk for developing a Vitamin B_12_ deficiency, which is often mitigated through a form of Vitamin B_12_ supplementation [67]. As dietary supplements were not incorporated into the final nutrient analysis, B_12_ intake from non-food sources may provide further insight into the deviance in estimates of Vitamin B_12_ intake from Diet ID™ and NDSR.

The findings from this analysis support and expand upon the results from a previous study comparing Diet ID™ to Automated Self-Administered 24 h (ASA24) dietary recalls [36]. The Nutritious Eating with Soul (NEW Soul) study was a 2-year randomized nutrition intervention aimed at comparing the impact of two dietary patterns on the risk of cardiovascular disease among African American adults [36]. Although study populations differed in population size (NEW Soul *n* = 68), age (NEW Soul = 50 ± 9.6 years), and race/ethnicity (NEW Soul = 100% African American), the findings for diet quality, as measured by HEI-2015, as well as cholesterol, potassium, Vitamin C, and Vitamin E were significant between Diet ID™ and both ASA24 and NDSR dietary recalls in their respective study populations. Findings from the NEW Soul study observed significant associations in carbohydrate and protein intake, as well as copper, Vitamin B_1_, and Vitamin B_12_, some of which were not observed in the present analysis [36]; however, it should be noted that the NEW Soul study analyzed mean nutrient intake by aggregating values across all participants, whereas data analysis was performed comparing individual output from both devices in this study. Thus, comparing the magnitude of significance between studies may not be feasible as the statistical approaches were not in congruence.

Furthermore, interviewer-administered dietary recalls are considered a higher quality assessment tool for capturing dietary intake data compared to self-administered dietary recalls due to the methodical probing to acquire exact dietary details [68]. While ASA24 dietary recalls are less participant and researcher burdensome, NDSR dietary recalls are considered to be a more rigorous dietary assessment tool [69]. However, ASA24 and NDSR dietary assessments have limitations in both time and resources; therefore, Diet ID™ may be an alternative tool that can capture similar nutrient output rapidly and with vastly reduced participant and researcher burden. In addition to the more rigorous dietary collection method used in this study, the inclusion of objective measures of skin carotenoid scores measured via the Veggie Meter^®^ and plasma carotenoids further promotes the use of Diet ID™ to measure nutrient intake, specifically those associated with fruit and vegetable consumption. The advantages of Diet ID™ have the ability to advance dietary intake assessment methodology, though it should be noted that Diet ID™ was designed to measure overall dietary patterns and alternative assessments may be recommended to calculate exact nutrient amounts, kinetics, or degradation of dietary compounds.

Innovative techniques to successfully capture the intricacies of dietary intake are needed to reduce participant and researcher burden in the research setting, as well as extend beyond research to improve dietary monitoring for public health benefit [70,71]. Dietary intake is closely associated with chronic disease risk, and dietary habits are often established prior to adulthood [72]. College students are a unique category of emerging adults, as many individuals in this life stage are making food choices independently for the first time, drastically altering their eating behaviors [73]. Most recently, the average HEI-2015 score for US adults was 58 out of a maximum score of 100 [74], and it has been observed that diet quality further decreases in the college student population due to financial limitations in affording healthy foods and environmental barriers to access [75]. Diet ID™ and NDSR were strongly correlated for predicting HEI-2015 scores (τ = 0.55, *p* < 0.0001); however, it should be noted that the level of agreement between the two measurements becomes less strong at HEI-2015 scores above 80 with a deviation of 7.14%. For this reason, assessing dietary intake in this population presents challenges that are often difficult to capture using traditional dietary assessment methods.

Despite these challenges, Diet ID™ was able to quickly estimate diet quality, consumption of total calories, protein, carbohydrates, and a majority of micronutrients, phytonutrients, and nutrients of concern with substantially less participant and researcher burden than other established methodologies, which signals potential for Diet ID™ to be utilized in clinical and outpatient settings as a dietary assessment method. Additionally, as the image-based technology allows for universal visual recognition, Diet ID™ may be able to be implemented in populations of low or limited literacy, and non-native English speakers. This study assessed the use of Diet ID™ in a population of college students, including individuals experiencing acute and chronic food insecurity.

### 4.3. Strengths and Limitations

It is imperative to recognize both the strengths and limitations of the present study. This study is the first to compare the innovative Diet ID™ technology to subjective and objective measures of dietary intake in a population of emerging adults. As this is a secondary validation from the previously mentioned study disrupted due to the COVID-19 pandemic, the total sample size was intended to be larger; however, the observed sample size in the data collected was sufficiently powered to analyze plasma carotenoids as the primary outcome. Thus, it is possible that the present study is underpowered to detect associations in certain nutrients of interest with high interindividual variability.

Due to the racial and ethnic diversity of the college student population at the University of California Davis, foods commonly consumed by participants may have not been present in the NDSR database nor in the images displayed in Diet ID™. To account for this, the Diet ID™ algorithm is currently expanding their patented algorithm to include a larger database of culturally diverse foods to better encompass the diversity of the eating patterns among people living in the US and to identify dietary patterns in other parts of the world. As Diet ID™ does not account for dietary supplements, all reported supplements were excluded from the NDSR nutrient output; therefore, intake of some nutrients may be higher than recorded as a result of supplementation. For the purpose of this analysis, dietary intake data without supplements was used for uniformity between outputs. While NDSR dietary recalls were unannounced and Diet ID™ was utilized as a self-assessment with limited supervision, it is possible that there was desirability or response bias among the participants. College students generally consume a lower quality diet than other adult populations; therefore, these findings may not be generalizable to all adult populations [76,77,78].

## 5. Conclusions

The findings from this study support the use of Diet ID™ as a rapid, non-invasive dietary assessment tool that may provide comparable estimates of nutrient consumption against repeated 24 h NDSR dietary recalls, skin carotenoid scores, and plasma carotenoids. Innovative diet capture technology, such as Diet ID™, has the potential to be implemented in both clinical and community settings to increase habitual dietary monitoring, with the goal of developing awareness around food choices to initiate health-promoting behaviors across the lifespan and in racially and ethnically diverse populations.

## Figures and Tables

**Table 1:** Baseline participant characteristics expressed as mean ± standard deviations for age, BMI, SCS, and nutrition knowledge, and the number and percentage of participants in subgroup by sex, race/ethnicity, and food security status (*n* = 48).

Age, Years (Mean ± SD)	22.09 ± 2.36
**Biological Sex**	
Male	12 (25%)
Female	36 (75%)
**Race/Ethnicity**	
African American/Black, not of Hispanic origin	1 (2%)
American Indian/Alaska native	0
Asian/Pacific Islander	23 (48%)
White, not of Hispanic origin	9 (19%)
Latin/Hispanic (Mexican-American, Puerto Rican, Cuban)	10 (21%)
Other	1 (2%)
Unknown/Prefer not to answer	4 (8%)
**Food Security Status**	
High	19 (40%)
Marginal	15 (31%)
Low	8 (17%)
Very Low	6 (12%)
**BMI (mean ± SD; kg/m^2^)**	
Total	24.58 ± 5.04
Male	25.79 ± 4.47
Female	24.18 ± 5.22
Timepoint 1: SCS (mean ± SD)	322.98 ± 114.42
Timepoint 2: SCS (mean ± SD)	341.35 ± 113.98

**Table 2:** Correlation coefficients between nutrient values predicted by Diet ID™ and 24 h dietary recalls (*n* = 42) by Pearson’s correlation^a^ or Kendall’s tau correlation ^b^.

Nutrient	Correlation Coefficient	*p*-Value
HEI-2015 Score ^b^	0.55	**<0.001**
Calories (kcals) ^a^	0.36	**0.02**
Protein (g) ^a^	0.55	**0.0002**
Carbohydrates (g) ^a^	0.31	**<0.05**
Fat (g) ^a^	0.29	NS (*p* = 0.06)
Cholesterol (mg) ^b^	0.32	**0.003**
Vitamin A (mcg) ^a^	0.39	**0.01**
Total Carotenoids (mcg) ^a^	0.44	**0.003**
α-carotene (mcg) ^b^	0.14	NS (*p* = 0.19)
β-carotene (mcg) ^b^	0.39	**0.0003**
Lycopene (mcg) ^b^	−0.09	NS (*p* = 0.40)
Lutein and Zeaxanthin (mcg) ^a^	0.58	**0.0001**
Dietary Fiber (g) ^a^	0.64	**<0.0001**
Calcium (mg) ^a^	0.36	**0.02**
Vitamin C (mg) ^a^	0.44	**0.003**
Vitamin D (mcg) ^a^	0.13	NS (*p* = 0.41)
Vitamin E (mg) ^a^	0.35	**0.02**
Sodium (mg) ^a^	0.36	**0.02**
Potassium (mg) ^a^	0.58	**0.0001**
Folate (mcg) ^a^	0.37	**0.02**
Iron (mg) ^a^	0.31	**0.04**
Vitamin B_1_ (Thiamin) (mg) ^a^	0.13	NS (*p* = 0.40)
Vitamin B_2_ (Riboflavin) (mg) ^a^	0.34	**0.03**
Vitamin B_3_ (Niacin) (mg) ^a^	0.42	**0.005**
Vitamin B_6_ (Pyridoxine) (mg) ^a^	0.57	**0.0001**
Vitamin B_12_ (Cobalamin) (mcg) ^b^	0.18	NS (*p* = 0.09)

^a^ Calculated using Pearson’s correlation coefficient (*ρ*). ^b^ Calculated using Kendall’s tau (τ).

**Table 3:** Relationship between skin carotenoid scores (SCS) measured using the Veggie Meter^®^ and plasma carotenoids measured using Diet ID™ controlling for BMI (*n* = 42).

Variables	Linear Regression (Adjusted R^2^)	*p*-Value
SCS and total carotenoids from Diet ID™; controlling for BMI	0.41	**<0.0001**
Total plasma carotenoids and Total Carotenoids from Diet ID™; controlling for BMI	0.37	**0.0001**
SCS and total plasma carotenoids, controlling for BMI	0.68	**<0.0001**

## Data Availability

Data, analytical code, and research materials described in this study will be made available from the corresponding author upon request, pending application and approval.

## Supplementary Materials

The following supporting information can be downloaded at: https://www.mdpi.com/article/10.3390/nu15020409/s1, File S1: Bland–Altman’s Plot Analysis.
