# Supplementary material for: Validation of Diet ID™ in Predicting Nutrient Intake Compared to Dietary Recalls, Skin Carotenoid Scores, and Plasma Carotenoids in University Students
Source: Nutrients. 2023 Jan 13;15(2):409. doi: 10.3390/nu15020409 (PMC9865232; doi:10.3390/nu15020409)

Figure S1. Agreement between NDSR and Diet ID for HEI-2015 Score

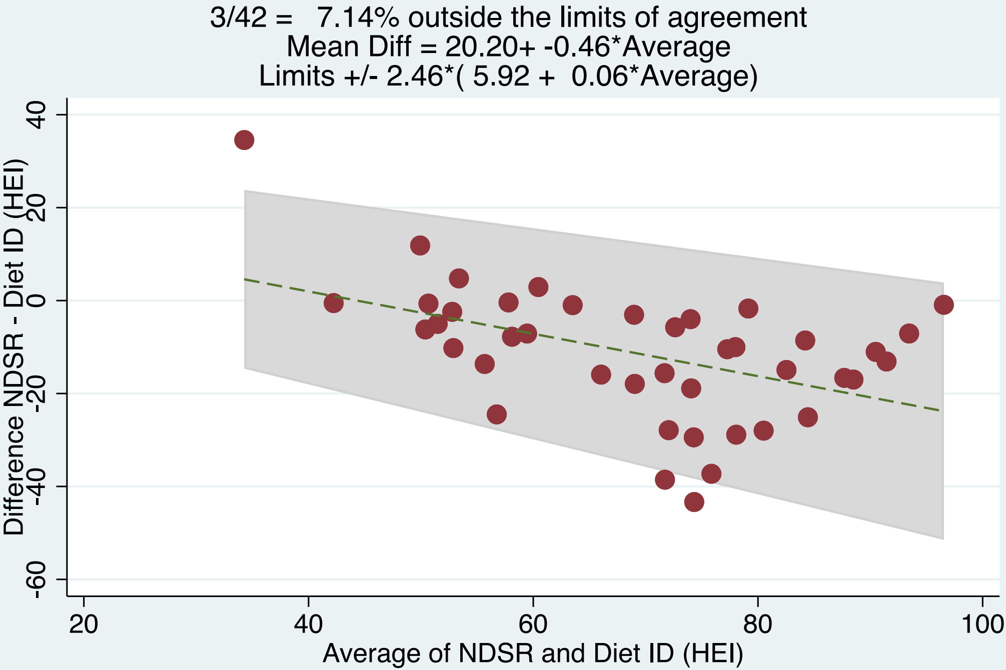

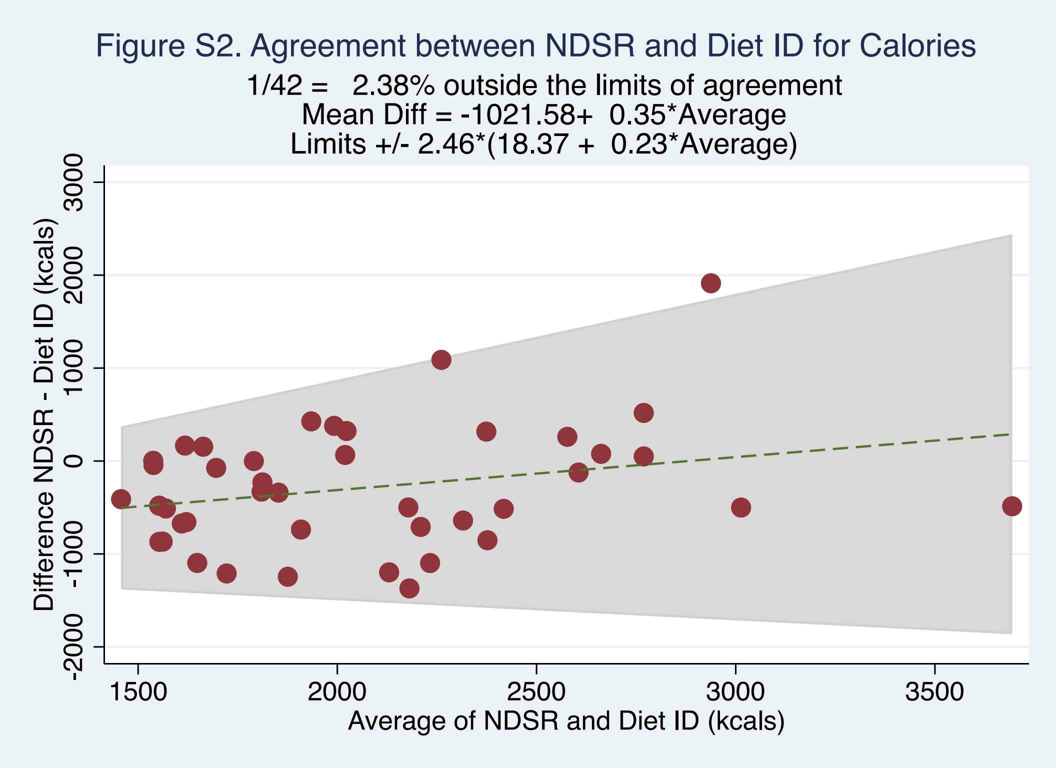
Figure S2. Agreement between NDSR and Diet ID for Calories
1/42 = 2.38% outside the limits of agreement
Mean Diff = -1021.58+ 0.35*Average
Limits +/- 2.46*(18.37 + 0.23*Average)
Difference NDSR - Diet ID (kcals)
-2000
-1000
0
1000
2000
3000
1500
2000
2500
3000
3500
Average of NDSR and Diet ID (kcals)

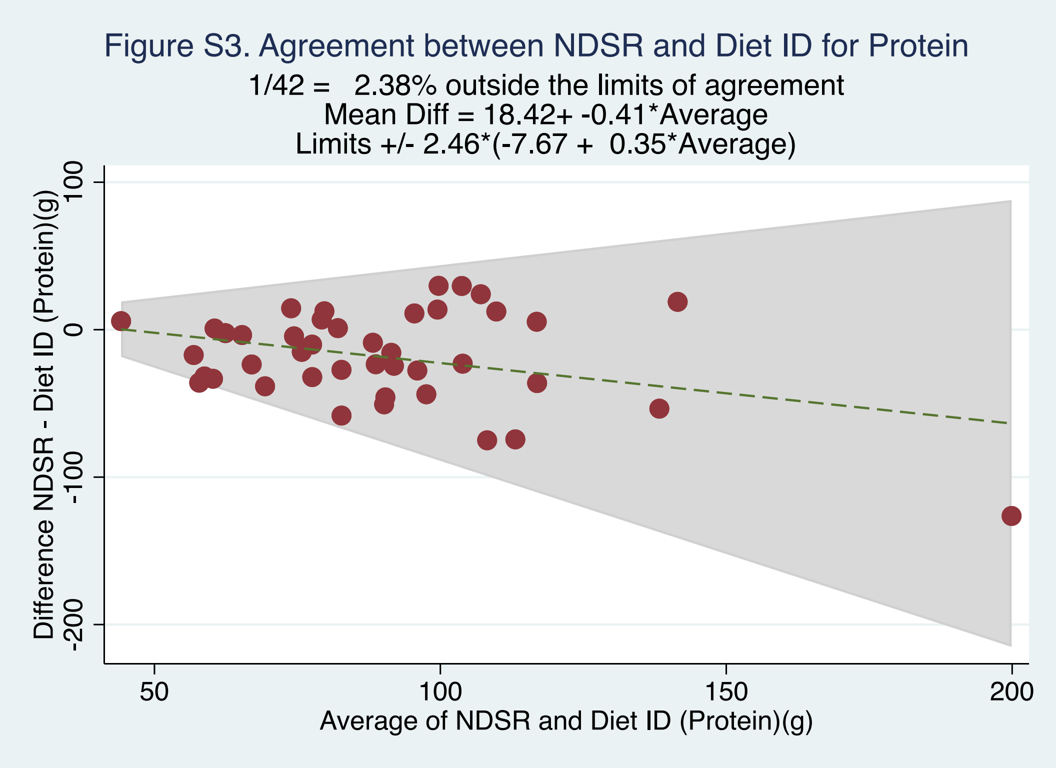
Figure S3. Agreement between NDSR and Diet ID for Protein
1/42 = 2.38% outside the limits of agreement
Mean Diff = 18.42+ -0.41*Average
Limits +/- 2.46*(-7.67 + 0.35*Average)
Difference NDSR - Diet ID (Protein)(g)
100
0
-100
-200
50
100
150
200
Average of NDSR and Diet ID (Protein)(g)

Figure S4. Agreement between NDSR and Diet ID for Carbohydrates

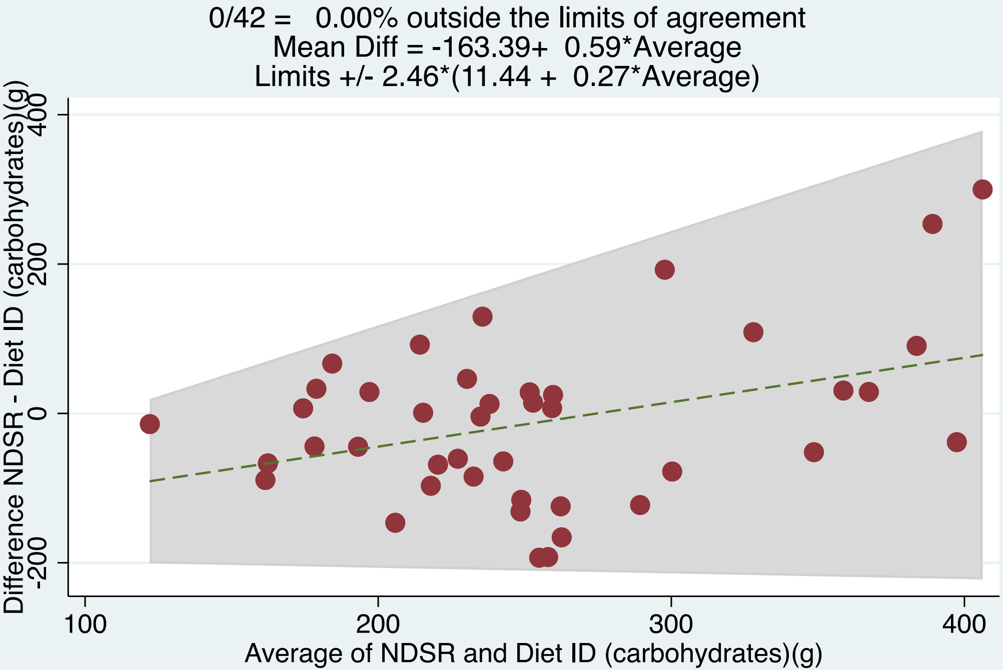

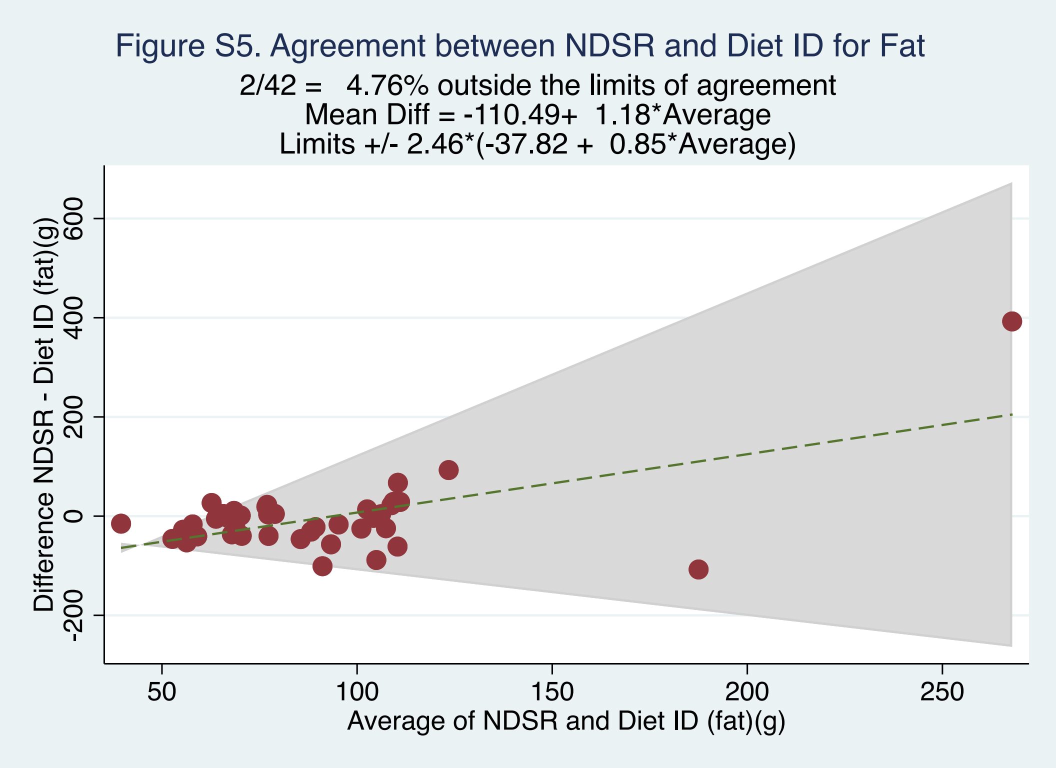
Figure S5. Agreement between NDSR and Diet ID for Fat
2/42 = 4.76% outside the limits of agreement
Mean Diff = -110.49+ 1.18*Average
Limits +/- 2.46*(-37.82 + 0.85*Average)
Difference NDSR - Diet ID (fat)(g)
600
400
200
0
-200
50
100
150
200
250
Average of NDSR and Diet ID (fat)(g)

Figure S6. Agreement between NDSR and Diet ID for Cholesterol

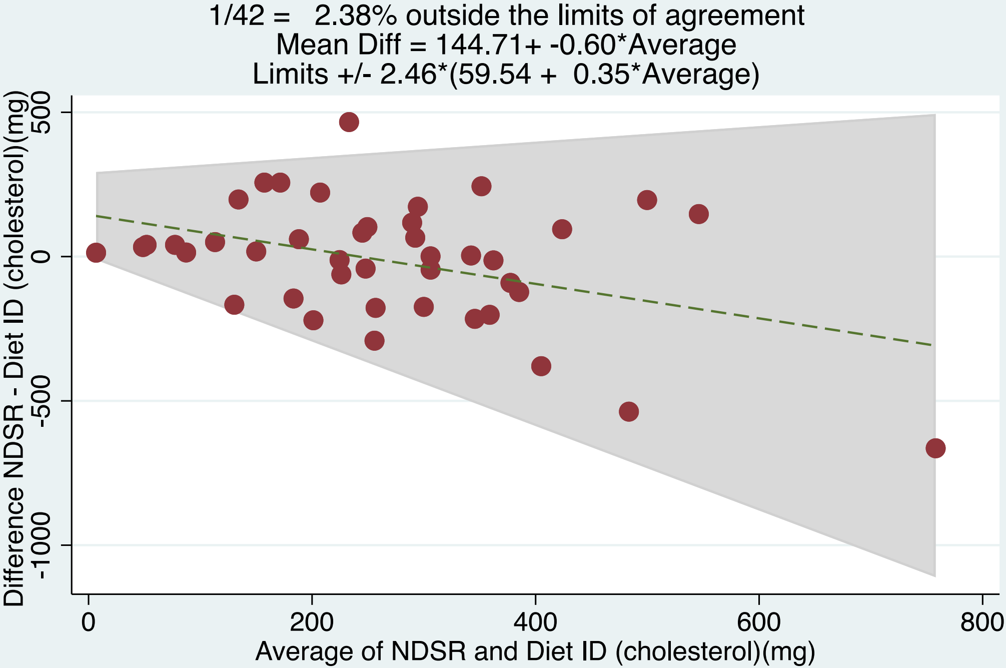

Figure S7. Agreement between NDSR and Diet ID for Vitamin A

2/42 = 4.76% outside the limits of agreement
Mean Diff = -323.40+ 0.02*Average
Limits +/- 2.46*(253.52 + 0.25*Average)

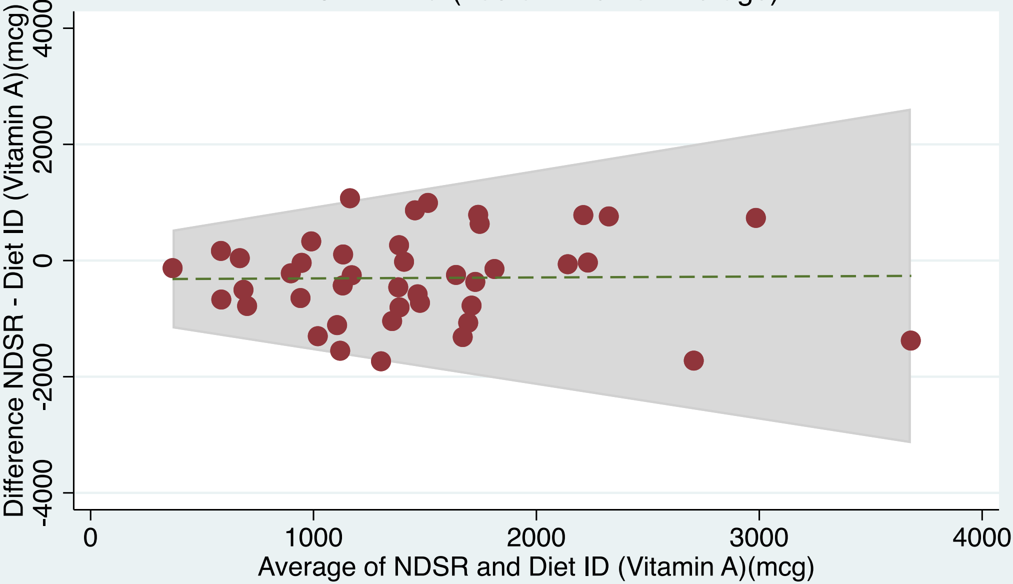

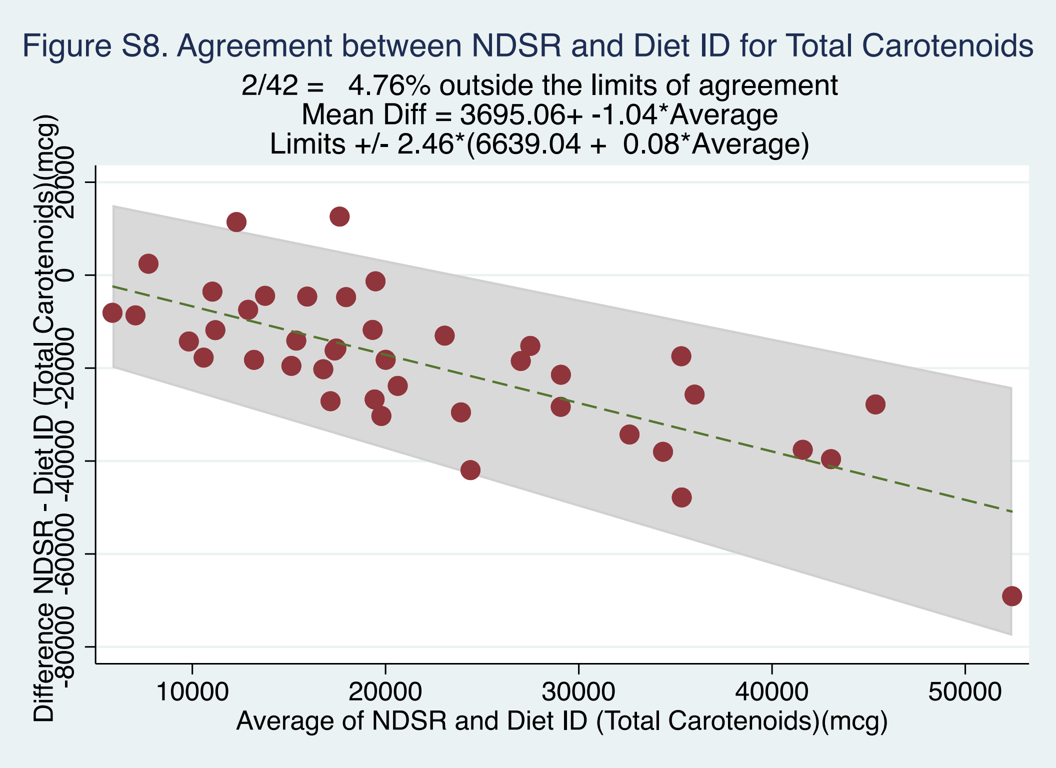
Figure S8. Agreement between NDSR and Diet ID for Total Carotenoids
2/42 = 4.76% outside the limits of agreement
Mean Diff = 3695.06+ -1.04*Average
Limits +/- 2.46*(6639.04 + 0.08*Average)
Difference NDSR - Diet ID (Total Carotenoids)(mcg)
-80000
-60000
-40000
-20000
0
20000
10000
20000
30000
40000
50000
Average of NDSR and Diet ID (Total Carotenoids)(mcg)

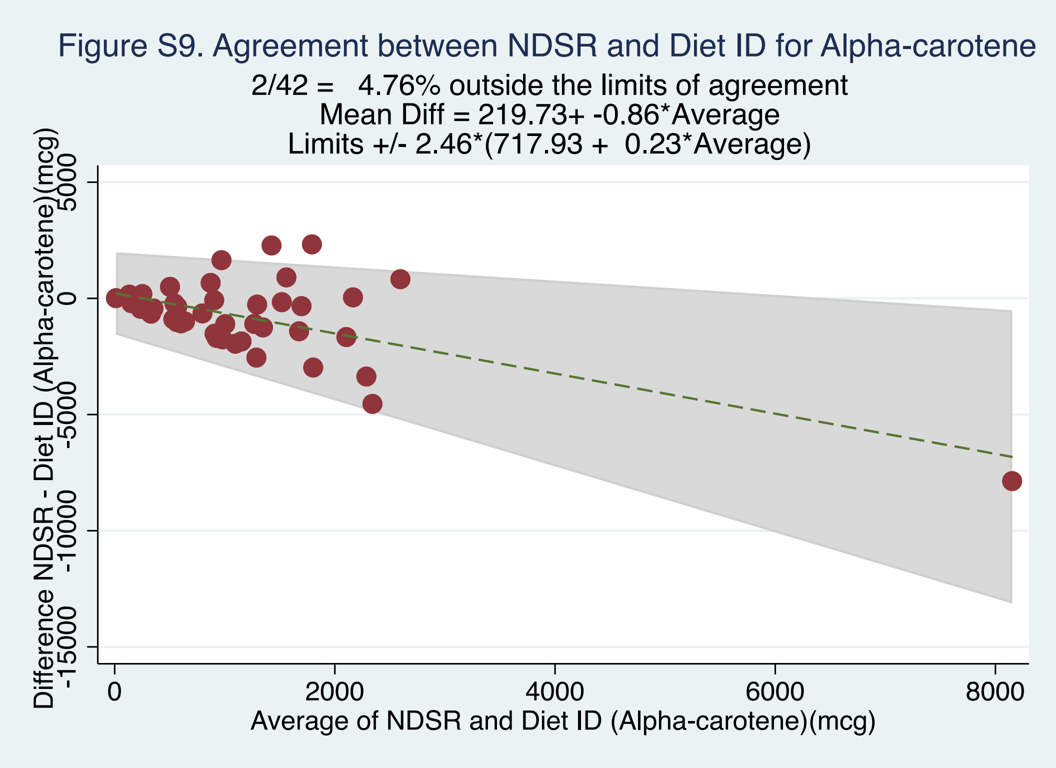
Figure S9. Agreement between NDSR and Diet ID for Alpha-carotene
2/42 = 4.76% outside the limits of agreement
Mean Diff = 219.73+ -0.86*Average
Limits +/- 2.46*(717.93 + 0.23*Average)
Difference NDSR - Diet ID (Alpha-carotene)(mcg)
5000
0
-5000
-10000
-15000
0
2000
4000
6000
8000
Average of NDSR and Diet ID (Alpha-carotene)(mcg)

Figure S10. Agreement between NDSR and Diet ID for Beta-carotene

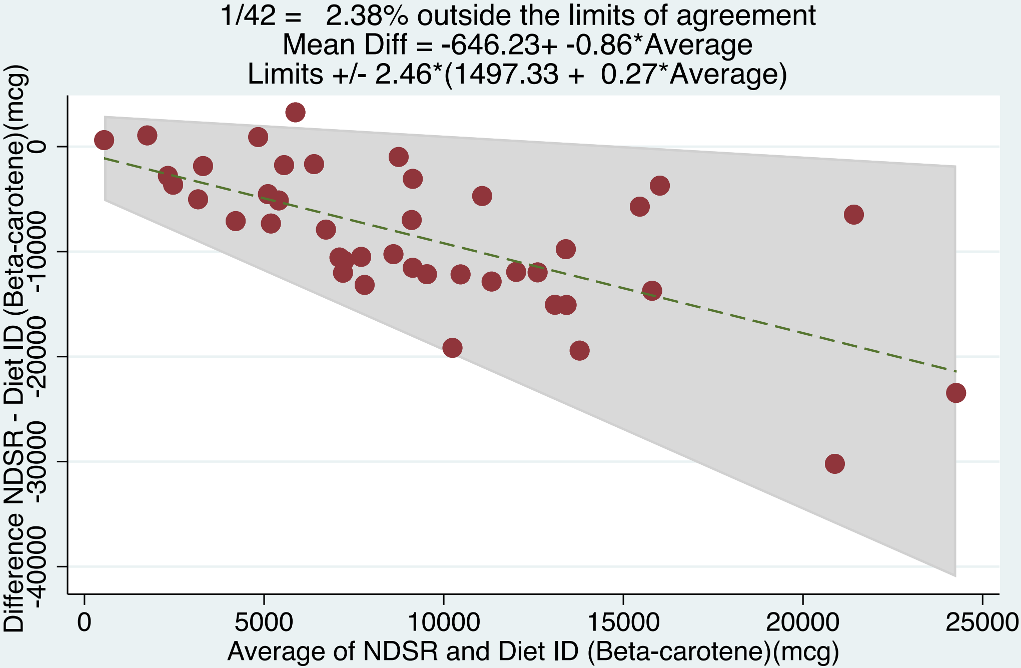

Figure S11. Agreement between NDSR and Diet ID for Lycopene

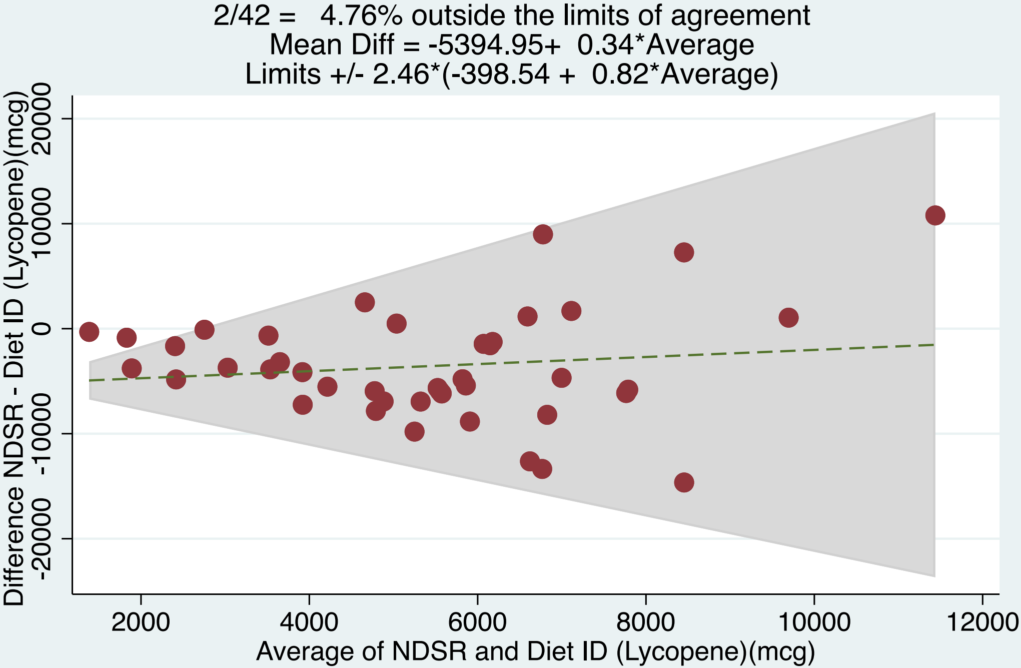

Figure S12. Agreement between NDSR and Diet ID for Lutein & Zeaxanth

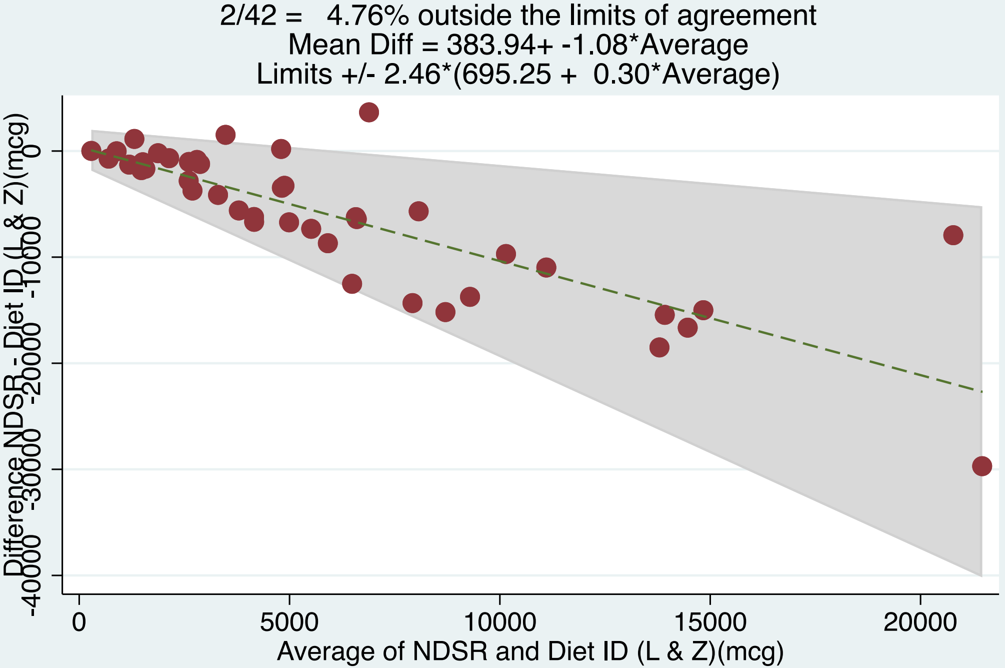

Figure S13. Agreement between NDSR and Diet ID for Fiber

0/42 = 0.00% outside the limits of agreement
Mean Diff = -56.36+ 1.47*Average
Limits +/- 2.46*(15.78 + 0.42*Average)

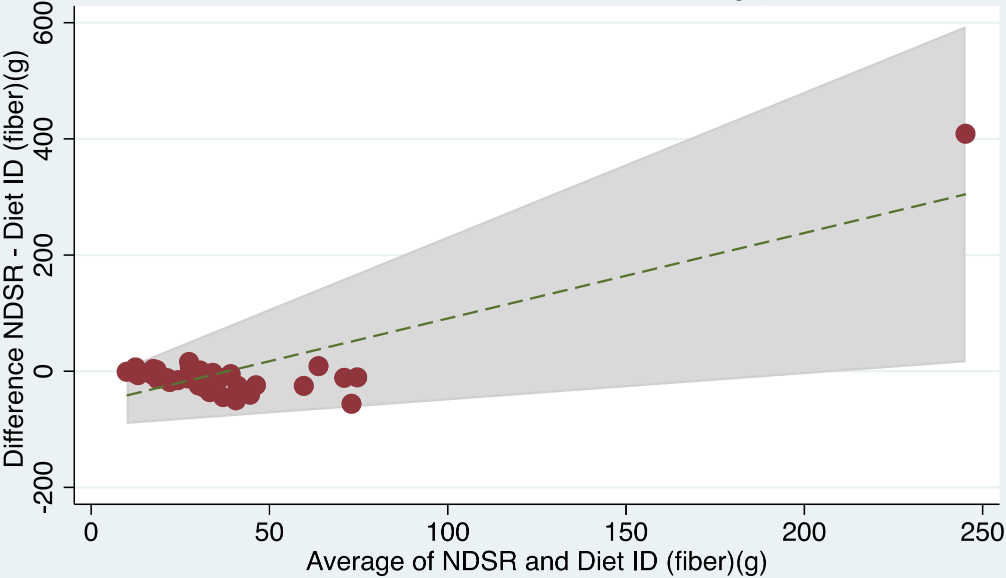

Figure S14. Agreement between NDSR and Diet ID for Calcium

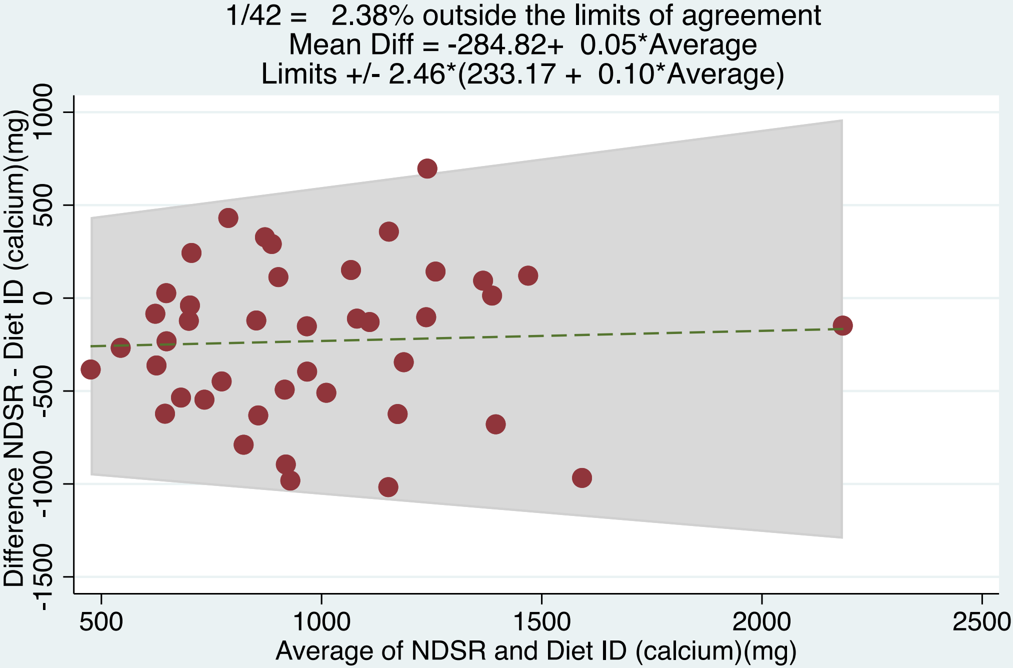

Figure S15. Agreement between NDSR and Diet ID for Vitamin C

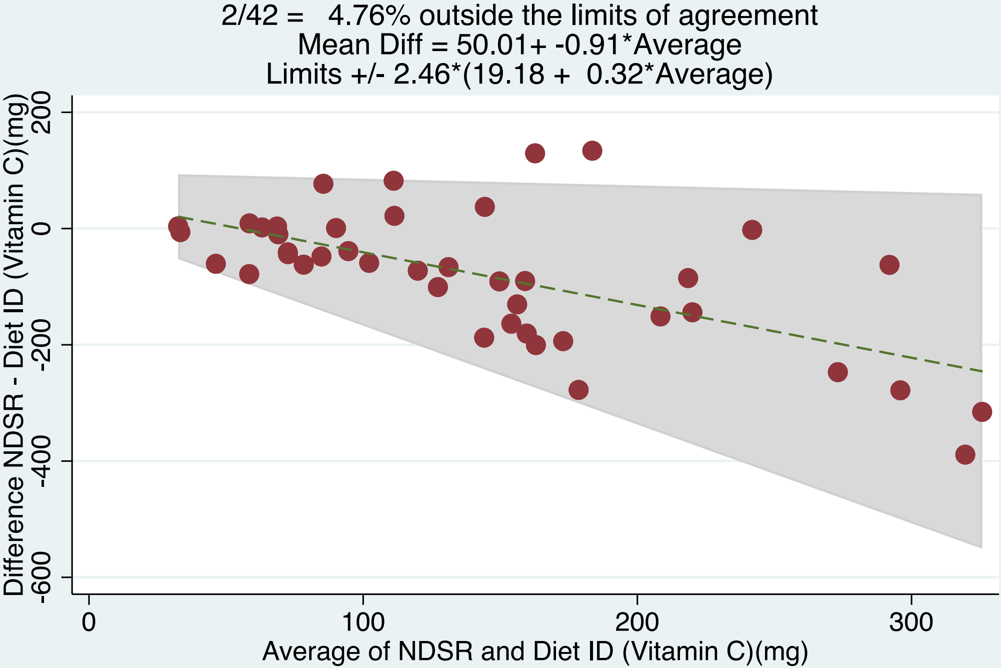

Figure S16. Agreement between NDSR and Diet ID (Vitamin D)

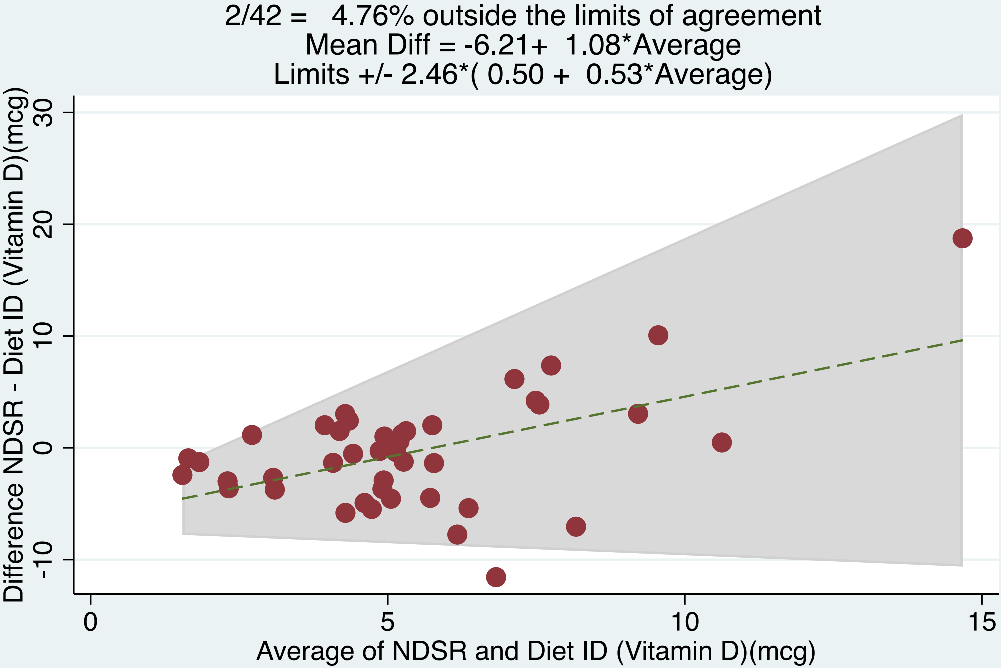

Figure S17. Agreement between NDSR and Diet ID for Vitamin E

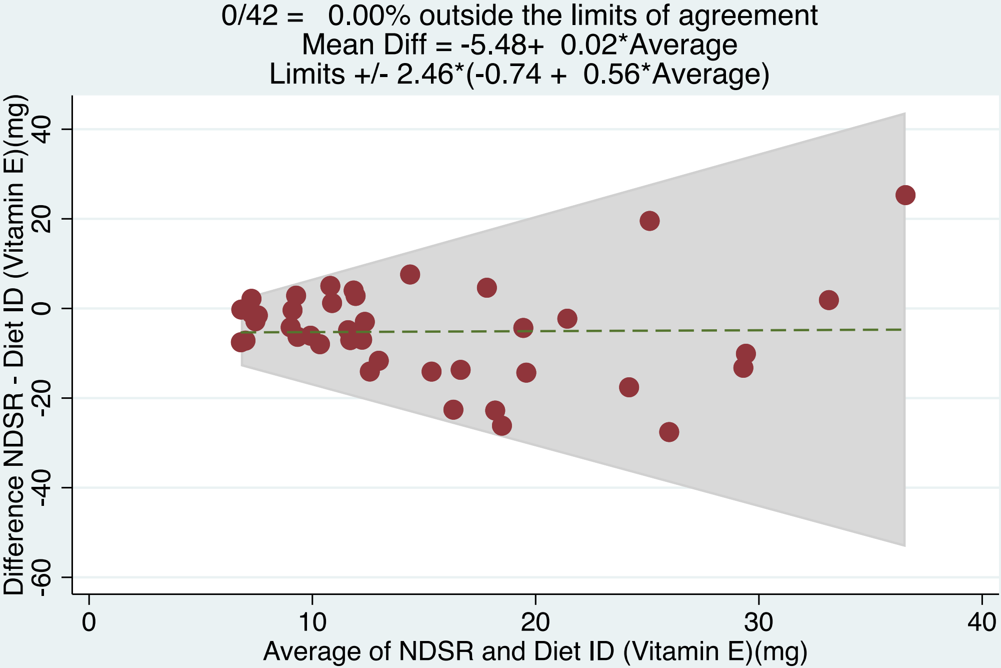

Figure S18. Agreement between NDSR and Diet ID (Sodium)

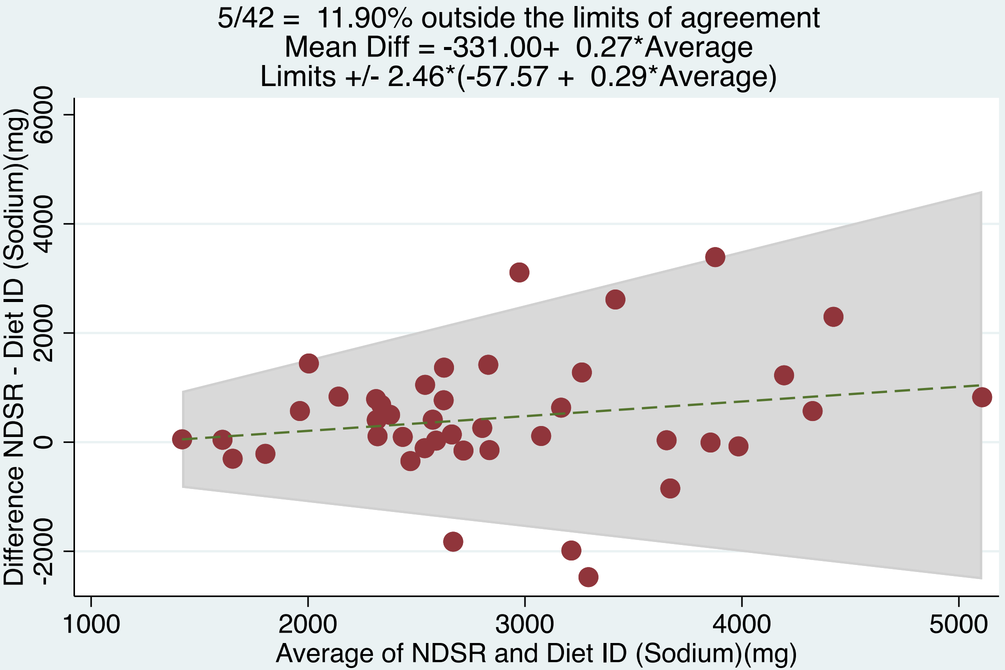

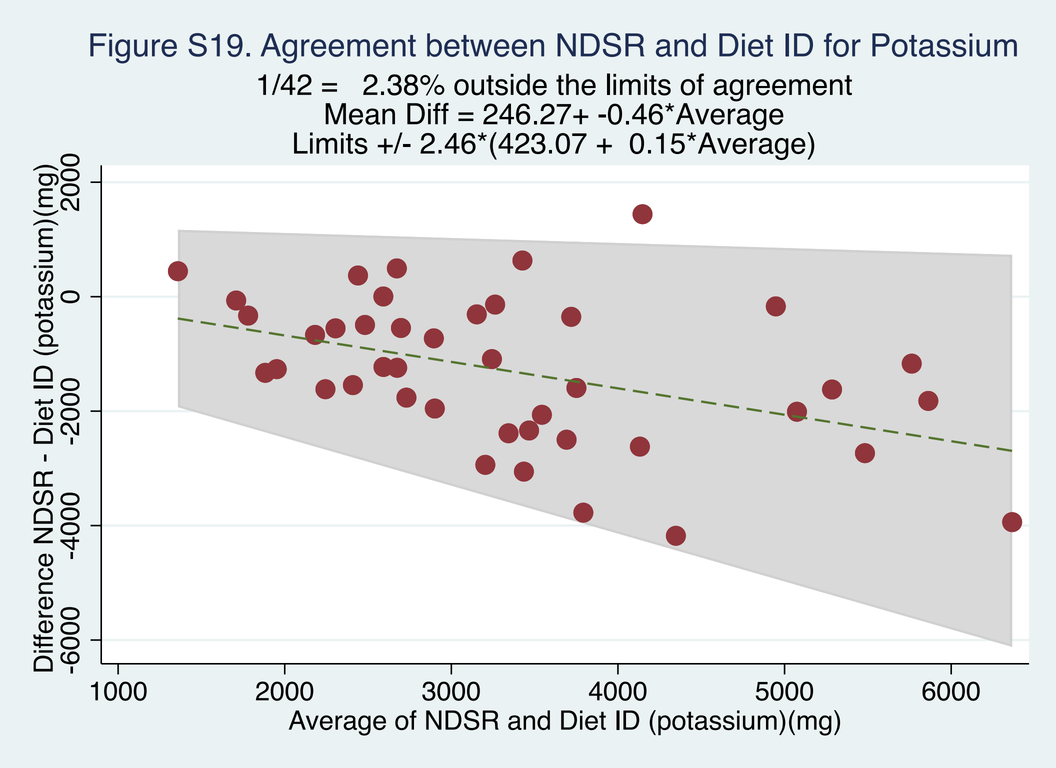
Figure S19. Agreement between NDSR and Diet ID for Potassium
1/42 = 2.38% outside the limits of agreement
Mean Diff = 246.27+ -0.46*Average
Limits +/- 2.46*(423.07 + 0.15*Average)
Difference NDSR - Diet ID (potassium)(mg)
2000
0
-2000
-4000
-6000
1000
2000
3000
4000
5000
6000
Average of NDSR and Diet ID (potassium)(mg)

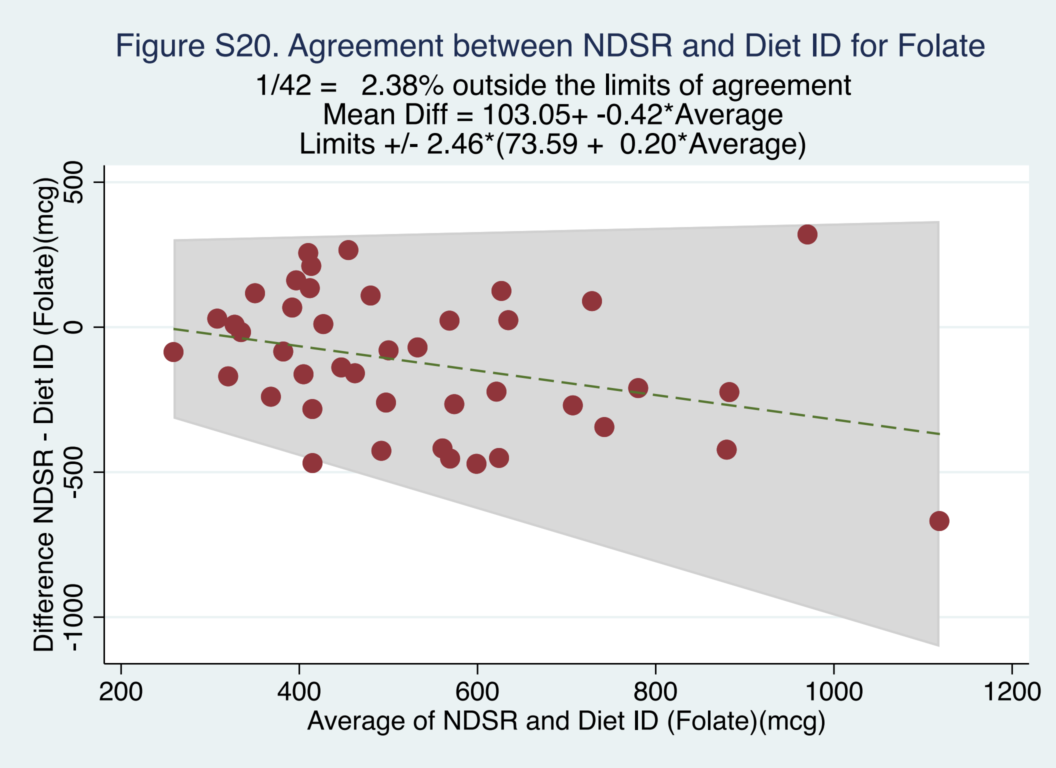
Figure S20. Agreement between NDSR and Diet ID for Folate
1/42 = 2.38% outside the limits of agreement
Mean Diff = 103.05+ -0.42*Average
Limits +/- 2.46*(73.59 + 0.20*Average)
Difference NDSR - Diet ID (Folate)(mcg)
500
0
-500
-1000
200
400
600
800
1000
1200
Average of NDSR and Diet ID (Folate)(mcg)

Figure S21. Agreement between NDSR and Diet ID for Iron

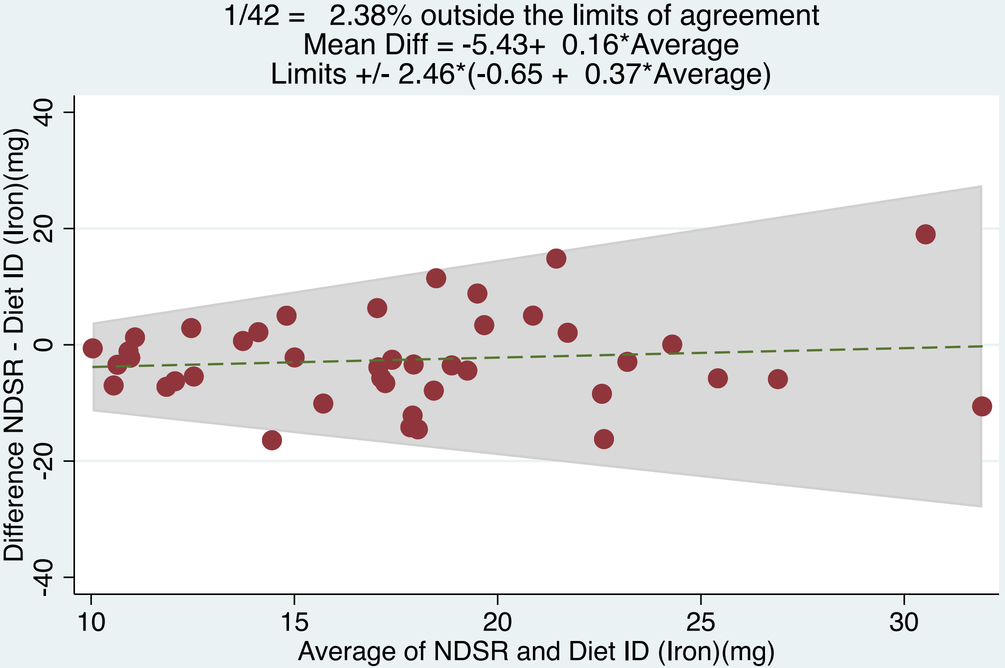

Figure S22. Agreement between NDSR and Diet ID for Vitamin B1

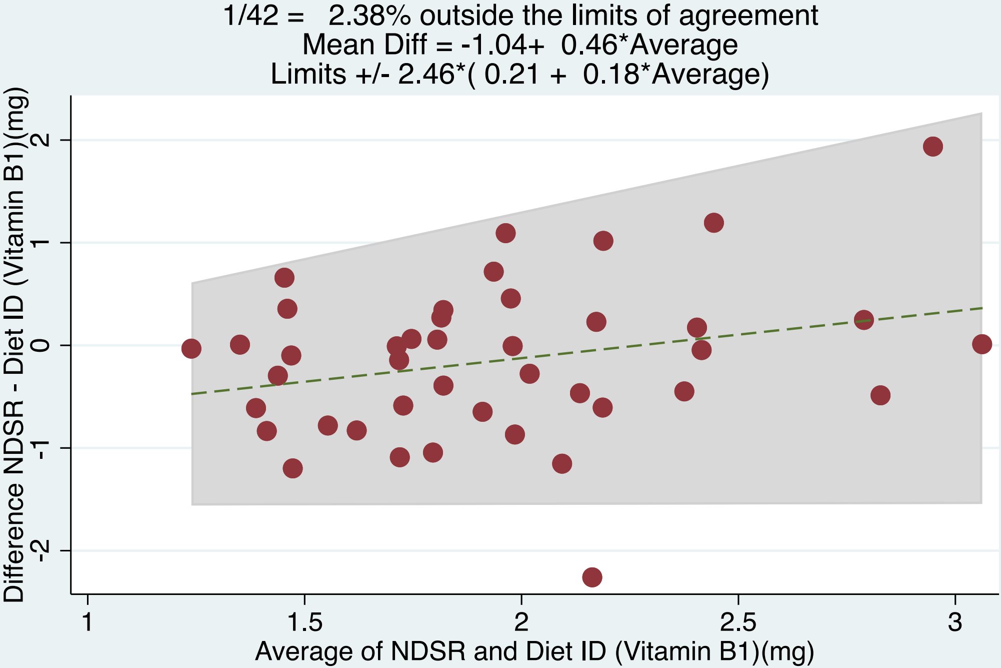

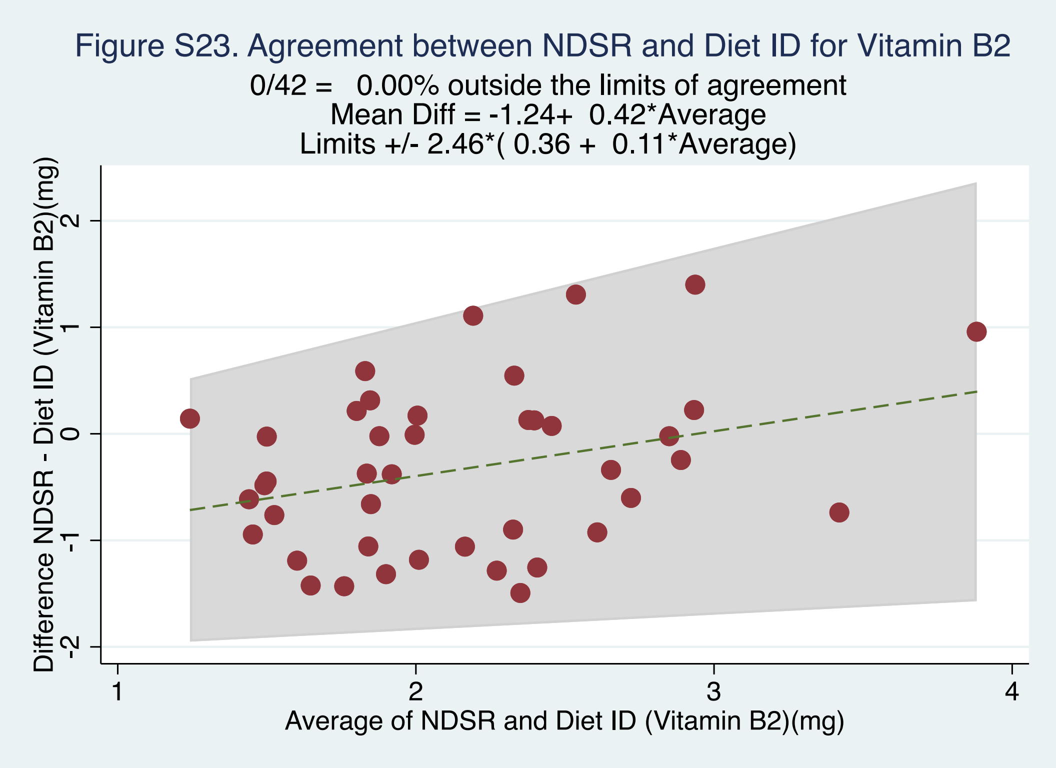
Figure S23. Agreement between NDSR and Diet ID for Vitamin B2
0/42 = 0.00% outside the limits of agreement
Mean Diff = -1.24+ 0.42*Average
Limits +/- 2.46*(0.36 + 0.11*Average)
Difference NDSR - Diet ID (Vitamin B2)(mg)
-2
-1
0
1
2
1
2
3
4
Average of NDSR and Diet ID (Vitamin B2)(mg)

Figure S24. Agreement between NDSR and Diet ID for Vitamin B3

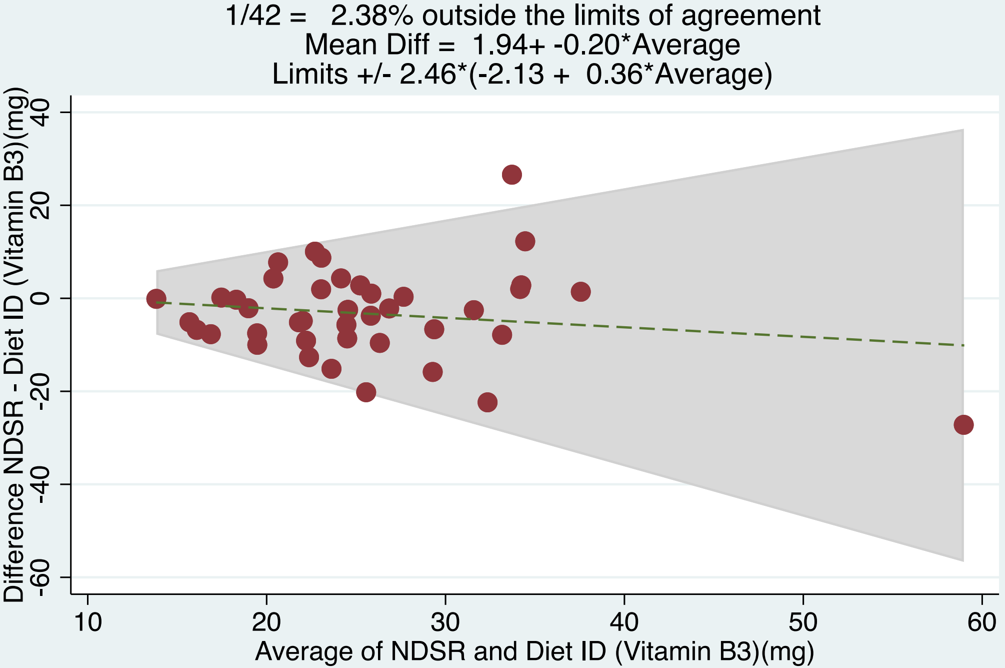

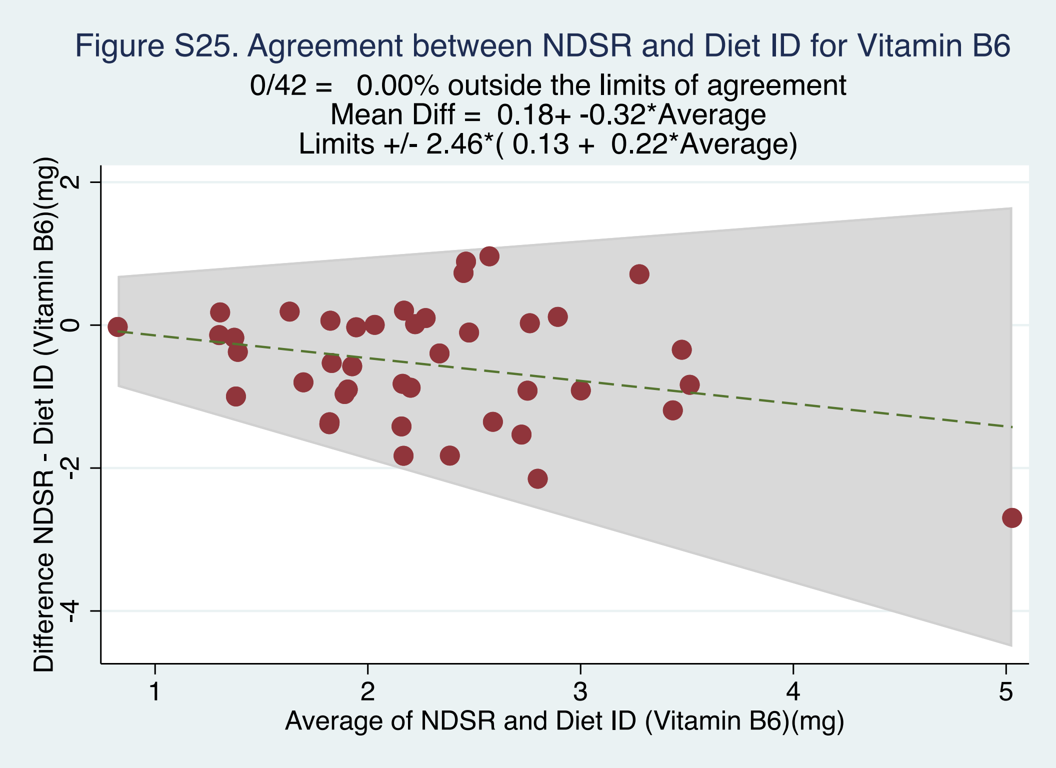

Figure S25. Agreement between NDSR and Diet ID for Vitamin B6
0/42 = 0.00% outside the limits of agreement
Mean Diff = 0.18+ -0.32*Average
Limits +/- 2.46*(0.13 + 0.22*Average)
Difference NDSR - Diet ID (Vitamin B6)(mg)
2
0
-2
-4
1
2
3
4
5
Average of NDSR and Diet ID (Vitamin B6)(mg)

Figure S26. Agreement between NDSR and Diet ID for Vitamin B12

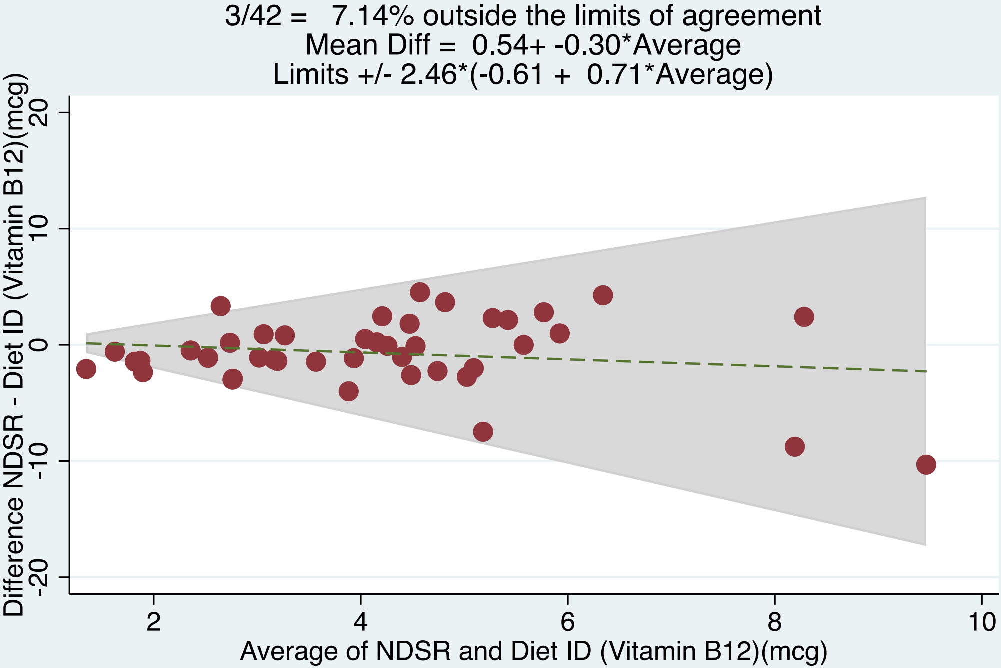

Supplement: Supplementary file 1 [file nutrients-15-00409-s001.zip › nutrients-2119117-supplementary.pdf]
